# Validation of the oncologic effect of hepatic resection for T2 gallbladder cancer: a retrospective study

**DOI:** 10.1186/s12957-018-1556-6

**Published:** 2019-01-07

**Authors:** Jin-Kyu Cho, Woohyung Lee, Jae Yool Jang, Han-Gil Kim, Jae-Myung Kim, Seung-Jin Kwag, Ji-Ho Park, Ju-Yeon Kim, Taejin Park, Sang-Ho Jeong, Young-Tae Ju, Eun-Jung Jung, Young-Joon Lee, Soon-Chan Hong, Chi-Young Jeong

**Affiliations:** 1 Department of Surgery, Gyeongsang National University Hospital, Gyeongsang National University Postgraduate School of Medicine, 79, Gangnam-ro, Jinju, 660-702 South Korea; 2 Department of Surgery, Gyeongsang National University Changwon Hospital, Gyeongsang National University Postgraduate School of Medicine, 11, Samjeongja-ro, Changwoun-si, 51472 South Korea

**Keywords:** Gallbladder carcinoma, Surgical strategies, Hepatic resection

## Abstract

**Background:**

While extended cholecystectomy is recommended for T2 gallbladder cancer (GBC), the role of hepatic resection for T2 GBC is unclear. This study aimed to identify the necessity of hepatic resection in patients with T2 GBC.

**Methods:**

Data of 81 patients with histopathologically proven T2 GBC who underwent surgical resection between January 1999 and December 2017 were enrolled from a retrospective database. Of these, 36 patients had peritoneal-side (T2a) tumors and 45 had hepatic-side (T2b) tumors. To identify the optimal surgical management method, T2 GBC patients were classified into the hepatic resection group (*n* = 44, T2a/T2b = 20/24) and non-hepatic resection group (*n* = 37, T2a/T2b = 16/21). The recurrence pattern and role of hepatic resection for T2 GBC were then investigated.

**Results:**

Mean age of the patients was 69 (range 36–88) years, and the male-to-female ratio was 42:39 (male, 51.9%; female, 48.1%). Hepatic-side GBC had a higher rate of recurrence than peritoneal-side GBC (44.4% vs. 8.3%, *p* = 0.006). The most common type of recurrence in T2a GBC was para-aortic lymph node recurrence (*n* = 2, 5.6%); the most common types of recurrence in T2b GBC were para-aortic lymph node recurrence (*n* = 7, 15.6%) and intrahepatic metastasis (*n* = 6, 13.3%). Hepatic-side GBC patients had worse survival outcomes than peritoneal-side GBC patients (76.0% vs. 96.6%, *p* = 0.041). Hepatic resection had no significant treatment effect in T2 GBC patients (*p* = 0.272). Multivariate analysis showed that lymph node metastasis was the only significant prognostic factor (*p* = 0.002).

**Conclusions:**

Hepatic resection is not essential for curative treatment in T2 GBC, and more systemic treatments are needed for GBC patients, particularly for those with T2b GBC.

## Background

Curative radical surgery is the gold standard treatment for gallbladder cancer (GBC) [1–3]. Depth of tumor invasion is crucial in determining the extent of resection. Simple cholecystectomy is the standard treatment modality for T1a GBC, while simple cholecystectomy or extended cholecystectomy is the optimal treatment for T1b GBC [4–7]. Meanwhile, extended cholecystectomy, including lymph node (LN) dissection and hepatic resection, is recommended for T2 GBC [4–6].

The 8th edition of the American Joint Committee on Cancer (AJCC) guidelines categorize T2 GBCs according to preoperative radiographic tumor location: peritoneal tumors are categorized as peritoneal-side (T2a) tumors and hepatic tumors are categorized as hepatic-side (T2b) tumors. This change in classification was based on the results of an international multicenter study that demonstrated worse survival outcomes in patients with hepatic-side tumors [8]. T2b GBC tumors show high incidence of nodal involvement and hepatic metastasis. By contrast, T2a GBC tumors have good prognosis with low rates of nodal and hepatic metastasis [9, 10]. Although there have been reports on the differences in the oncologic prognosis of T2a and T2b GBCs, no consensus has been reached on the survival benefit of hepatic resection for T2a and T2b GBCs.

This study aimed to investigate the role of hepatic resection in the treatment of T2 GBC, with a focus on the oncologic benefit of hepatic resection according to tumor location of T2 GBC.

## Methods

### Patients and ethical considerations

We retrospectively analyzed the medical data of patients who were pathologically diagnosed with T2 GBC and underwent curative resection between January 1999 and December 2017 at our hospital. The inclusion criteria were as follows: patients who underwent R0 resection and were pathologically diagnosed with T2 gallbladder adenocarcinoma. To determine preoperative T stage, all patients underwent radiological examinations, including abdominal computed tomography (CT) and/or ultrasonography. Patients who underwent incomplete resection (R1 and R2 resection), those who did not have preoperative radiographic images, those who had distant metastasis, those who had other pathologically diagnosed adenosquamous or small cell carcinomas, those who had comorbid malignancies, and those who were suspected of having preoperative T2 GBC but were found to have advanced disease based on intraoperative frozen section or histopathology findings were excluded. Finally, 81 patients with T2 GB adenocarcinoma, of which 36 were peritoneal-side (T2a) tumors and 45 were hepatic-side (T2b) tumors, who underwent R0 surgical resection were included. Clinicopathological data, including age, body mass index, sex, American Society of Anesthesiologists score, tumor markers, operation time, tumor size, tumor-node-metastasis (TNM) staging, hospital stay duration, complications, and adjuvant chemotherapy, were collected from medical records and analyzed. The pathologic TNM stage was defined according to the AJCC guidelines (8th edition) [11]. Pathologic T2 GBC was categorized based on the preoperative radiographic tumor location in the gallbladder, i.e., T2a as peritoneal and T2b as hepatic [8]. The patients were then divided into two groups according to the type of surgery: hepatic resection group (HR group, *n* = 44) and non-hepatic resection group (Non-HR group, *n* = 37). N1+ or N2 LN dissection was performed in T2 GBC patients. Para-aortic LN dissection and frozen biopsy were performed in selected patients who had enlarged LNs on preoperative radiologic examination. When positive para-aortic LN findings were observed in the frozen section, LN dissection was performed without hepatic resection. Recurrence patterns and survival outcomes were compared between the two groups. The site of recurrence was determined via radiographic imaging during follow-up. This retrospective study was approved by the Institutional Review Board of our hospital.

### Statistical analysis

The prognostic factors of survival in T2 GBC patients were investigated using univariate and multivariate Cox regression analyses. Disease-free survival (DFS) was calculated from the date of surgery to the date of recurrence. Cancer-specific overall survival (OS) was calculated from the date of surgery to the date of GBC-related death. Survival was calculated using the Kaplan-Meier method, and variables were analyzed using the log-rank test.

Pearson’s chi-square test or Fisher’s exact test and Student’s *t* test were used for comparisons between the two groups. Statistical analysis was performed using SPSS software version 22.0 (SPSS, Chicago, IL, USA), and a *p* value of < 0.05 was considered significant.

## Results

### Clinicopathological characteristics

A total of 81 GBC patients were included in the analysis. Among 81 patients who were pathologically diagnosed with T2 GBC, preoperative radiological examinations showed an accuracy of 82.7% (67/81) for T stage diagnosis. T stage was underestimated as T1 GBC in 9 patients and was overestimated as T3 GBC in 5 patients, based on preoperative radiologic examination results. The male-to-female ratio was 42:39 (male, 51.9%; female, 48.1%); mean age was 69 (range 36–88) years. Thirty-six (44.4%) and 45 (55.6%) patients were classified as having T2a (peritoneal-side) and T2b (hepatic-side) tumors, respectively. Clinicopathological characteristics of the patients according to hepatic resection are shown in Table 1. Forty-four patients (54.3%) underwent hepatic resection and 37 patients (45.7%) underwent non-hepatic resection. The patients underwent the following types of surgeries: simple cholecystectomy, 23; cholecystectomy with LN dissection, 13; cholecystectomy with hepatic resection and LN dissection, 38; cholecystectomy with extrahepatic bile duct resection (EHBD) and LN dissection, 1; and cholecystectomy with hepatic resection, EHBD, and LN dissection, 6 (Table 2). Hepatic resection, comprising of non-anatomical liver resection with a 3-cm resection margin from the cystic plate, was performed in 44 patients. None of the patients had any breach of tumor tissue while excising the gallbladder from the GB fossa. There were no significant differences in preoperative conditions, tumor markers, complications, tumor size, and T stage between the HR group and Non-HR group. However, the mean operation time and hospital stay duration were significantly shorter in the Non-HR group than in the HR group (342.5 ± 100.5 vs. 153.7 ± 102.3 min, *p* = < 0.001 and 13.3 ± 5.7 vs. 8.8 ± 8.8 days, *p* = 0.009, respectively; Table 2).

**Table 1:** Clinicopathological characteristics of the patients

Variable	Hepatic resection (*n* = 44)*	Non-hepatic resection (*n* = 37)*	*p* value
Age	64.9 ± 10.0	70.6 ± 9.8	0.012
Sex (M:F)	23:21	19:18	> 0.999
BMI (kg/m^2^)	22.5 ± 2.9	23.1 ± 3.1	0.407
ASA (1/2/3/4)	3/30/11/0	1/19/16/1	0.157
Combined GB stone	7 (15.9%)	9 (24.3%)	0.407
CEA (ng/mL)	4.1 ± 6.1	4.6 ± 8.1	0.780
CA19-9 (U/mL)	58.9 ± 105.5	38.2 ± 90.1	0.431
Operation time (min)	342.5 ± 100.5	153.7 ± 102.3	< 0.001
Complication rate	10 (22.7%)	6 (16.2%)	0.579
Clavien-Dindo classification (I, II, IIIa/IIIb, IV, V)	10/0 (22.7%/0%)	5/1 (13.5%/2.7%)	0. 323
Tumor size (mm)	30.7 ± 17.4	26.0 ± 16.2	0.213
T2a	20 (45.5%)	16 (43.2%)	> 0.999
T2b	24 (54.5%)	21 (56.8%)	
N0	28 (63.6%)	15 (40.5%)	< 0.001
N1	14 (31.8%)	6 (16.2%)	
N2	2 (4.5%)	0 (0%)	
Nx	0 (0%)	16 (43.2%)	
Adjuvant chemotherapy	23 (52.3%)	10 (27.0%)	0.025
Hospital stay (day)	13.3 ± 5.7	8.8 ± 8.8	0.009

*Data are presented as mean ± standard deviation for continuous data and percentages for categorical data. *BMI* body mass index, *ASA* American Society of Anesthesiologist physical status classification system, *CEA* carcinoembryonic antigen, *CA19-9* carbohydrate antigen 19-9

**Table 2:** Comparison of operation type

Type of operation	Hepatic resection (*n* = 44)	Non-hepatic resection (*n* = 37)	*p* value
Simple cholecystectomy	0 (0%)	23 (62.2%)	< 0.001
Cholecystectomy with LND	0 (0%)	13 (35.1%)	
Cholecystectomy with LND + HR	38 (86.4%)	0 (0%)	
Cholecystectomy with LND + EHBD	0 (0%)	1 (2.7%)	
Cholecystectomy with LND + HR + EHBD	6 (13.6%)	0 (0%)	

*LND* lymph node dissection, *HR* hepatic resection, *EHBD* extrahepatic bile duct resection

Adjuvant chemotherapy was administered to a greater number of patients in the HR group than that in the Non-HR group (52.3% vs. 27%, *p* = 0.025; Table 1). The HR group also had a significantly higher incidence of LN metastasis than the Non-HR group (*p* = < 0.001; Table 1).

### Recurrence pattern of T2 GBC

**Fig. 1:**
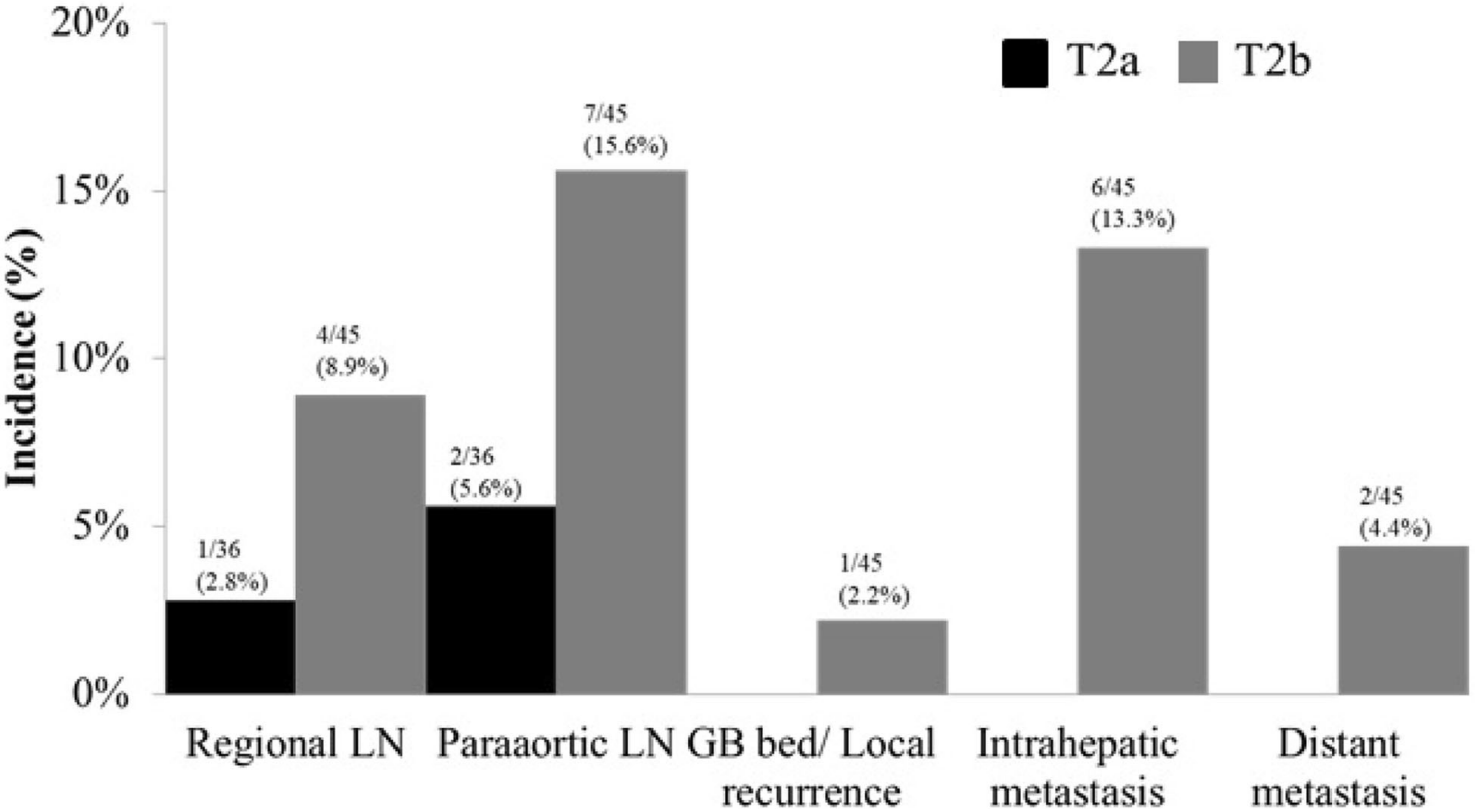
Recurrence pattern of T2 gallbladder carcinoma according to tumor location (*n* = 81). The incidences of recurrent site in T2 gallbladder carcinoma. There was a different recurrence pattern between T2a and T2b gallbladder carcinoma

The recurrence pattern of T2 GBC was significantly different between T2a and T2b tumors (Fig. 1). T2b GBC had a higher rate of recurrence than T2a GBC during follow-up (44.4% vs. 8.3%, *p* = 0.006). The most common types of recurrence in T2b GBC were para-aortic LN recurrence (*n* = 7, 15.6%) and intrahepatic metastasis (*n* = 6, 13.3%). In contrast, no intrahepatic metastasis occurred in patients with T2a GBC; only regional (*n* = 1, 2.8%) or para-aortic nodal recurrence (*n* = 2, 5.6%) was observed. Most recurrences were distant to the GB bed and comprised intrahepatic or bilateral intrahepatic lesions. Only one patient had GB perforation during cholecystectomy; this patient had a recurrent lesion near the GB bed.

### Survival in T2 GBC patients

**Fig. 2:**
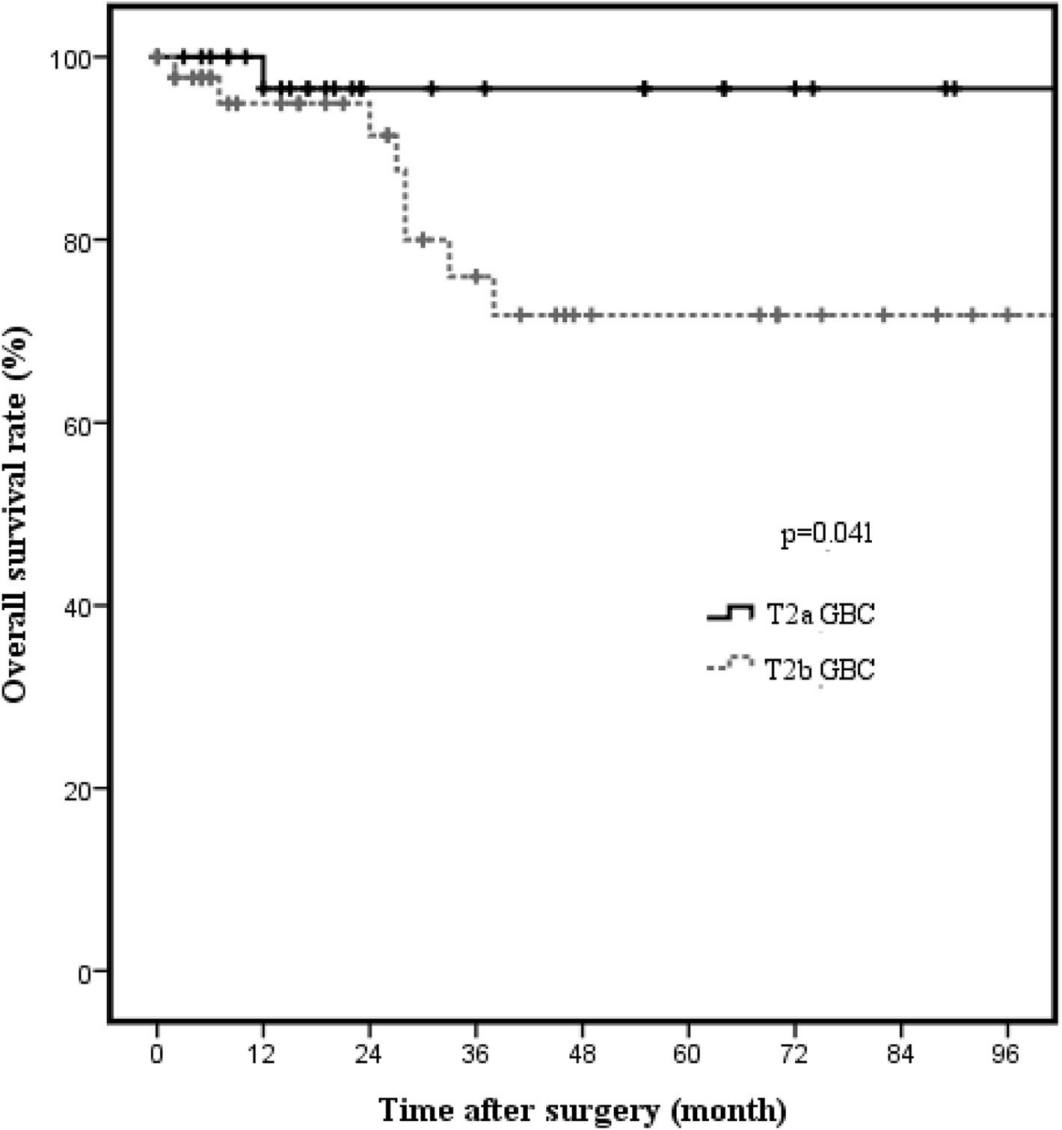
Overall survival rate in T2 gallbladder carcinoma according to tumor location (*n* = 81). The 3-year cancer-specific survival rate in patients with T2 gallbladder carcinoma was 96.6% in those with T2a gallbladder carcinoma and 76.0% in those with T2b gallbladder carcinoma. There was significant difference in survival according to tumor location (*p* = 0.041)

**Fig. 3:**
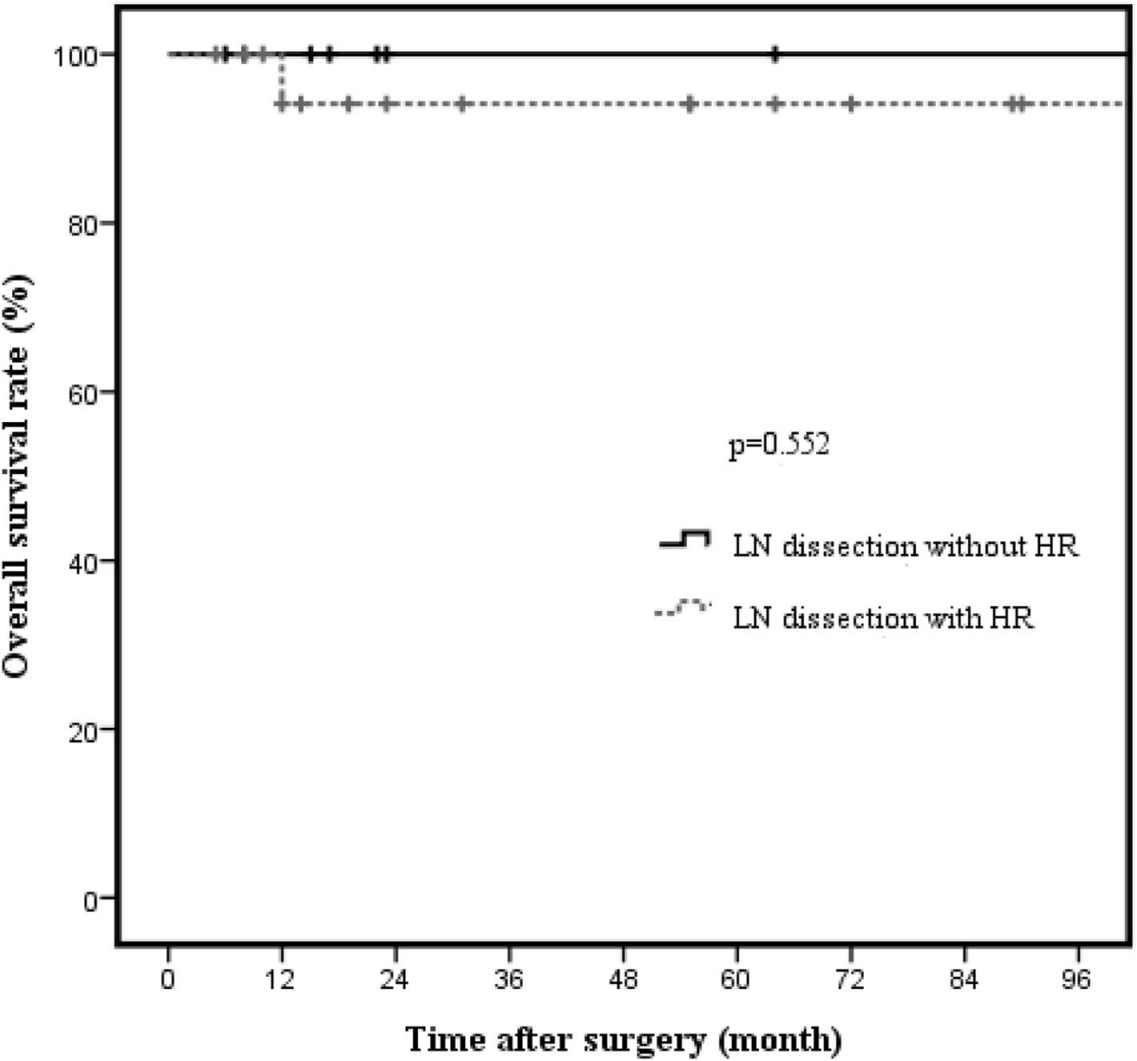
Overall survival rate in T2a gallbladder carcinoma according to hepatic resection (*n* = 28). The 3-year cancer-specific survival rates of T2a GBC with or without hepatic resection were 94.1% and 100%, respectively (*p* = 0.552)

**Fig. 4:**
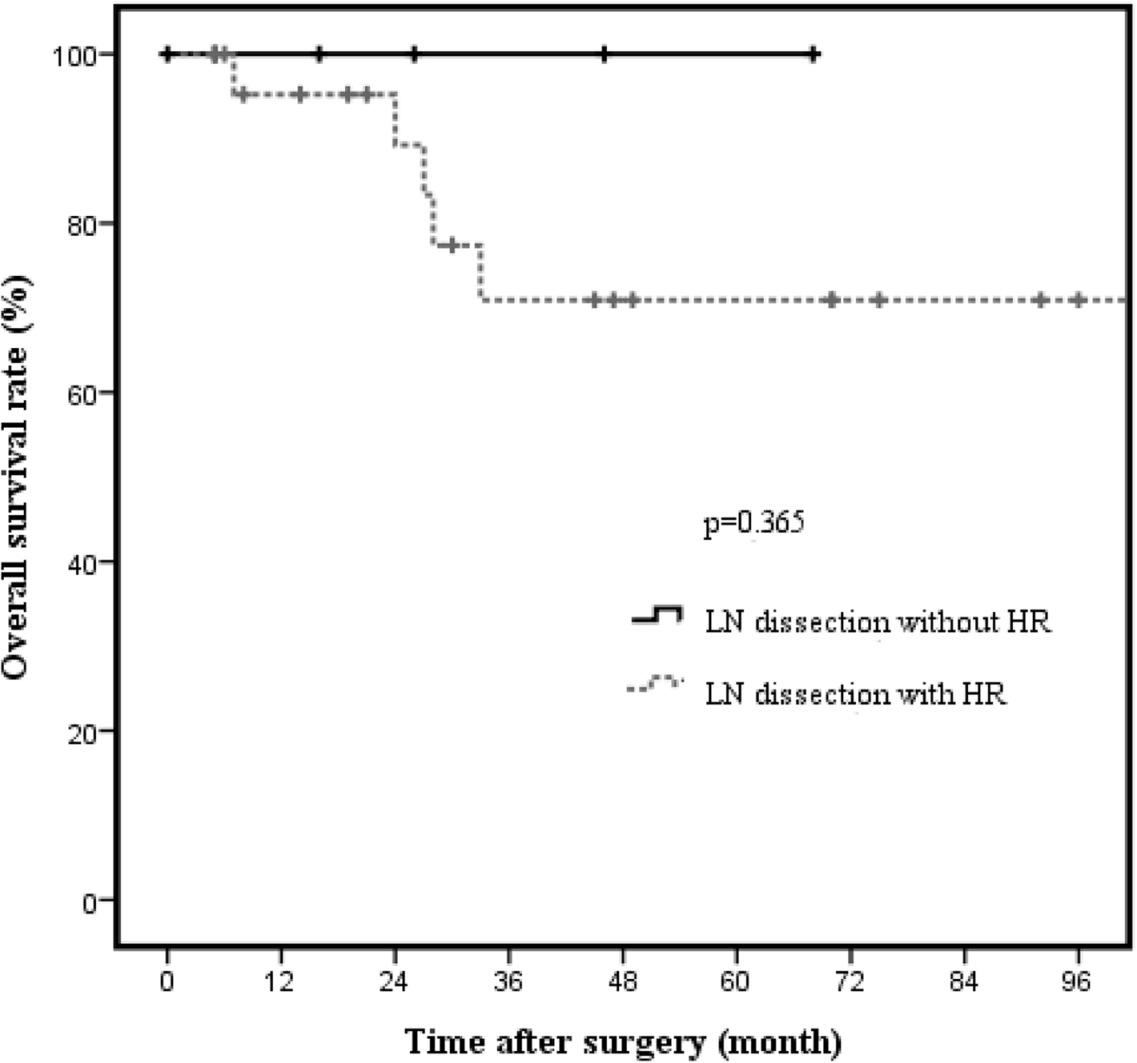
Overall survival rate in T2b gallbladder carcinoma according to hepatic resection (*n* = 30). The 3-year cancer-specific survival rates of T2b GBC with or without hepatic resection were 70.9% and 100%, respectively (*p* = 0.365)

Kaplan-Meier analysis showed significantly better survival of patients with T2a GBC (*n* = 36) than of patients with T2b GBC (*n* = 45) (96.6% vs. 76%, *p* = 0.041; Fig. 2). The 3-year OS rates were 96.6% and 76.0% in T2a GBC and T2b GBC patients, respectively. With respect to hepatic resection, no significant difference in OS rates were noted in both T2a GBC (94.1% vs. 100%, *p* = 0.552; Fig. 3) and T2b GBC (70.9% vs. 100%, *p* = 0.365; Fig. 4). No cancer-related deaths occurred during follow-up among T2 GBC patients who underwent LN dissection without hepatic resection. The 3-year OS rate was 100% in patients who underwent LN dissection without hepatic resection for T2a GBC and was 94.1% in the patients who underwent LN dissection with hepatic resection for T2a GBC. The 3-year OS rate was 100% in patients who underwent LN dissection without hepatic resection for T2b GBC and 70.9% in patients who underwent LN dissection with hepatic resection for T2b GBC.

**Fig. 5:**
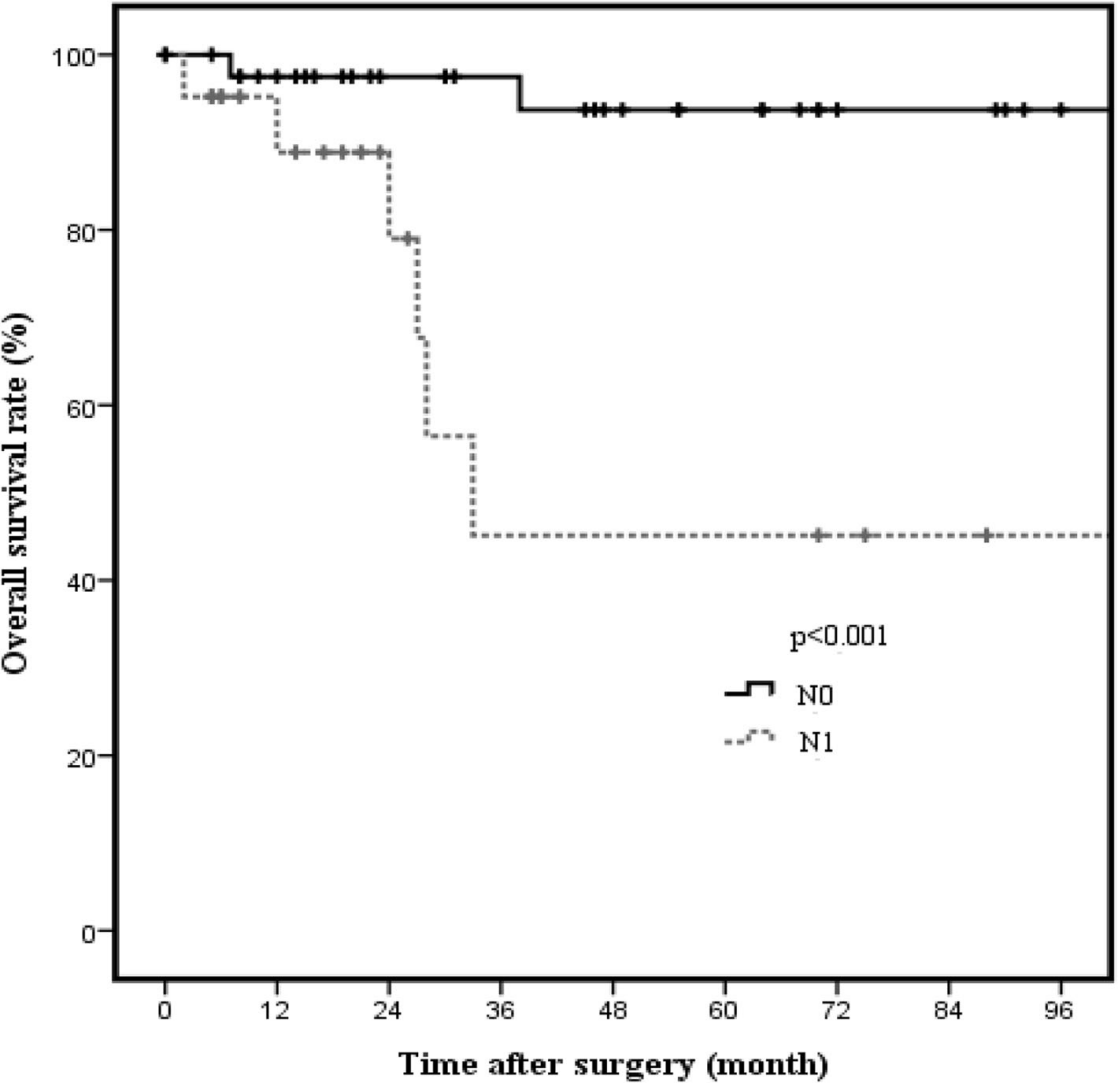
Overall survival rate of T2 GBC patients with or without lymph node metastasis (*n* = 81). The 3-year cancer-specific survival rates of patients with or without lymph node metastasis were 45.1% and 97.5%, respectively (*p* < 0.001)

**Fig. 6:**
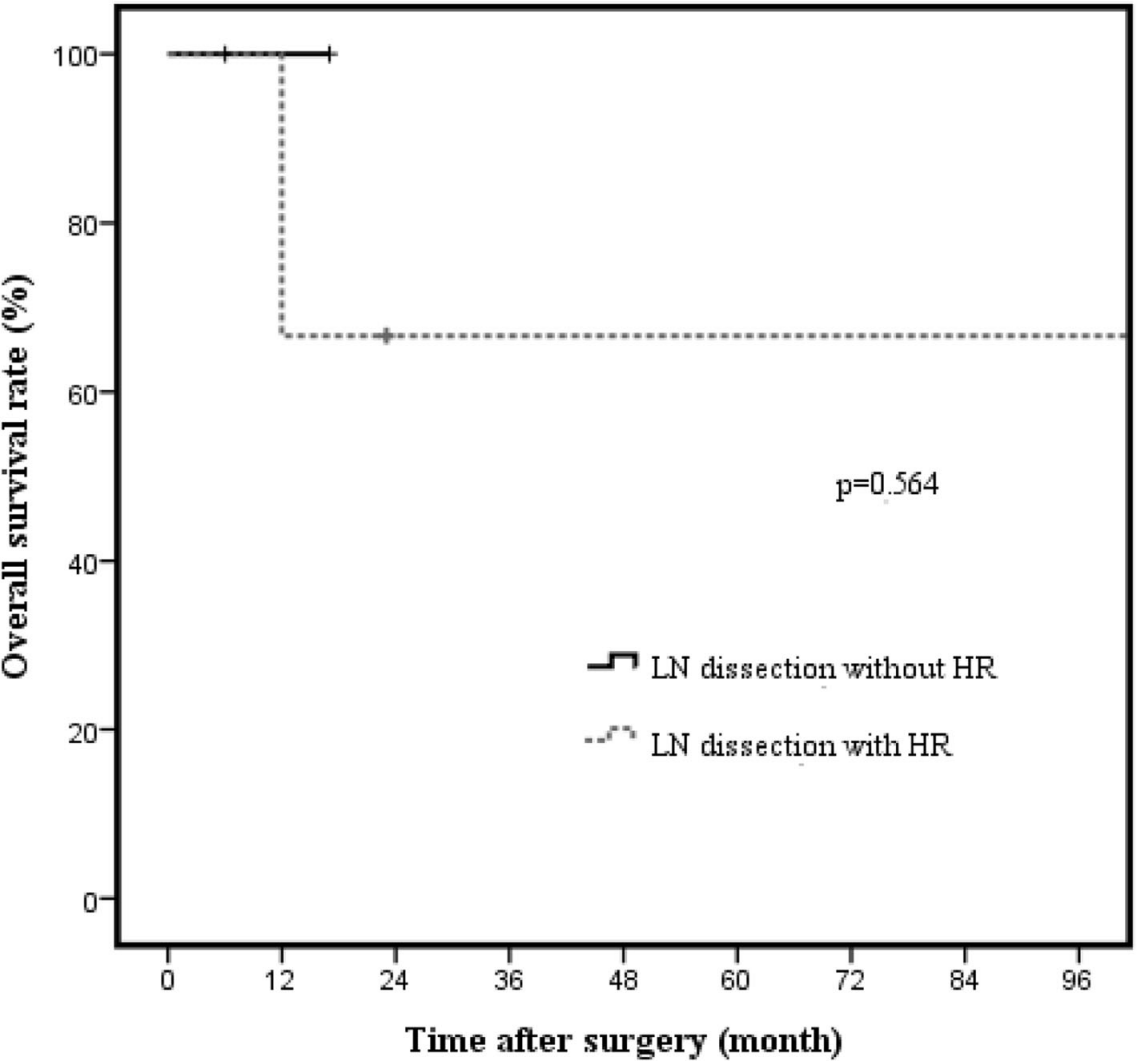
Cancer-specific overall survival rate in node-positive T2a gallbladder carcinoma according to hepatic resection (*n* = 5). The 3-year cancer-specific overall survival rate in node-positive T2a GBC between lymph node dissection without hepatic resection and lymph node dissection with hepatic resection were 66.7% and 100%, respectively (*p* = 0.564)

**Fig. 7:**
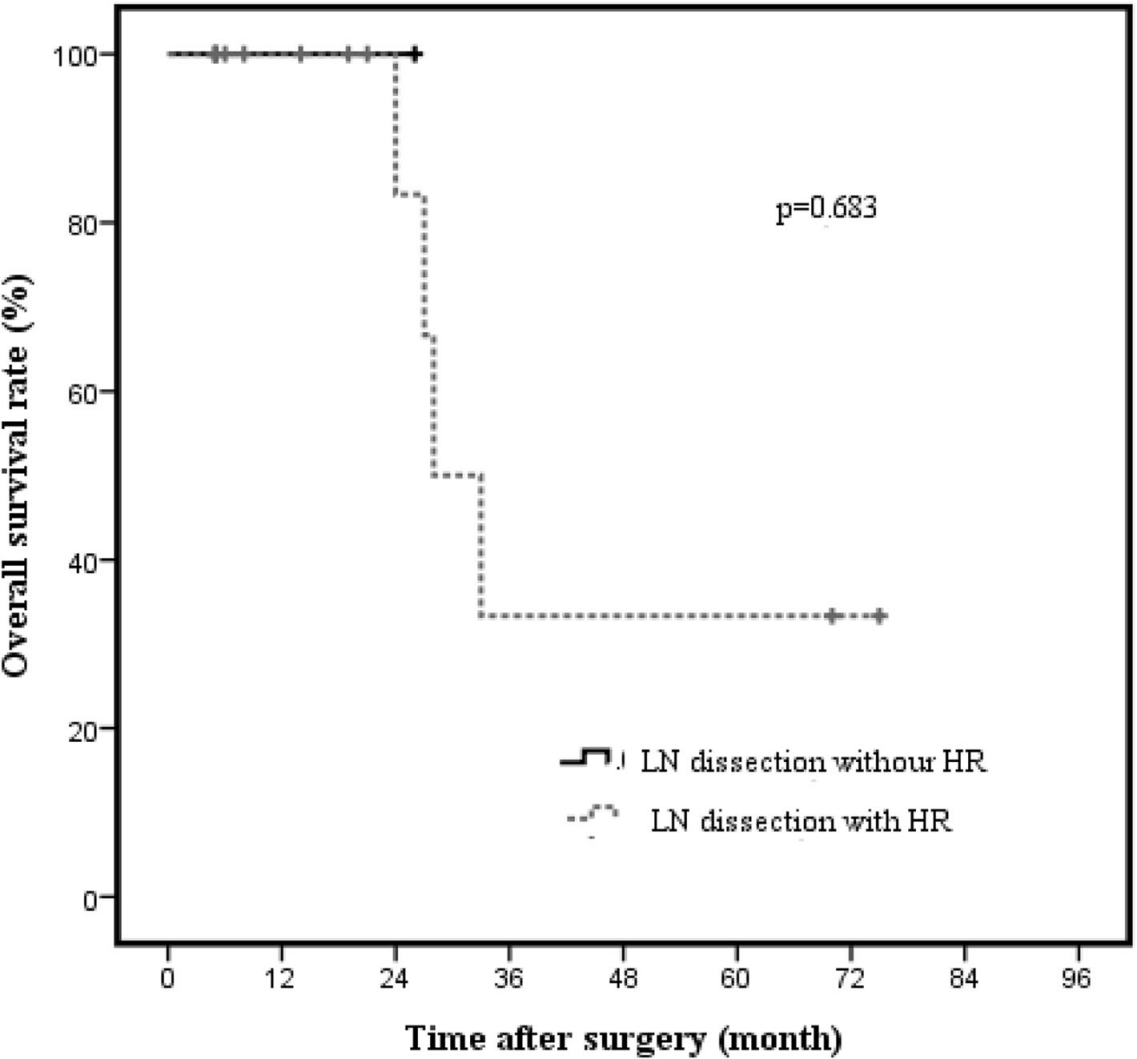
Cancer-specific overall survival rate in node-positive T2b gallbladder carcinoma according to hepatic resection (*n* = 15). The 3-year cancer-specific overall survival rate in node-positive T2b GBC between lymph node dissection without hepatic resection and lymph node dissection with hepatic resection were 33.3% and 100%, respectively (*p* = 0.683)

Kaplan-Meier analysis showed worse survival rates in patients with T2 GBC and LN metastasis than in those with T2 GBC without LN metastasis (*p* < 0.001; Fig. 5). In both T2a GBC with LN metastasis and T2b GBC with LN metastasis, there were no significant differences in OS rates in terms of hepatic resection (T2a GBC 66.7% vs. 100%, *p* = 0.564, Fig. 6; T2b GBC 33.3% vs. 100%, *p* = 0.683, Fig. 7).

### Prognostic factors of survival in T2 GBC patients

Results of univariate Cox regression analysis for identifying the prognostic factors of survival in T2 GBC patients are shown in Table 3. On univariate analysis, carcinoembryonic antigen (CEA) level (> 5 ng/mL), tumor location, and LN metastasis were significantly associated with poorer survival in T2 GBC patients. Meanwhile, LN metastasis was the only independent factor associated with survival in T2 GBC patients on multivariate Cox regression analysis (HR = 9.336, 95% CI = 2.295–37.985, *p* = 0.002). Hepatic resection was not a prognostic factor for survival in T2 GBC patients (HR = 1.246, 95% CI = 0.311–4.994, *p* = 0.756).

**Table 3:** Prognostic factors for T2 gallbladder cancer patients (*n* = 81)

Variables	Univariate analysis			Multivariate analysis		
	HR	95% CI	*p* value	HR	95% CI	*p* value
Female sex	1.403	0.376–5.232	0.614			
Age > 60 years	1.489	0.308–7.205	0.621			
Overweight (BMI > 25 kg/m^2^)	1.632	0.437–6.093	0.466			
CEA (> 5 ng/mL)	6.328	1.134–35.320	0.035	3.608	0.556–23.395	0.179
CA19-9 (> 37 U/mL)	26.762	0.010–68,340.593	0.412			
Further resection	0.700	0.175–2.801	0.614			
GB stone	1.625	0.203–13.005	0.647			
Tumor size (> 10 mm)	24.395	0.003–202,140.970	0.488			
T stage (T2a vs. T2b)	6.515	0.814–52.138	0.077	3.246	0368–28.624	0.289
Node metastasis	9.336	2.295–37.985	0.002	9.336	2.295–37.985	0.002
Complication	1.467	0.303–7.095	0.634			
Adjuvant chemotherapy	1.717	0.426–6.925	0.447			
Laparoscopic surgery	0.822	0.204–3.309	0.783	1.130	0.247–5.167	0.875

*BMI* body mass index, *GB* gallbladder, *CEA* carcinoembryonic antigen, *CA19-9* carbohydrate antigen 19-9

## Discussion

The surgical management involving LN dissection with GB bed resection for advanced GBC was introduced by Glenn [12]. Since then, hepatic resection has become the treatment of choice for GBC [4, 6].

Several studies have demonstrated improved outcomes with extended resection. However, all these studies were based on retrospective data of a small number of patients and had a low level of evidence. In addition, Kang et al. [13] reported a favorable survival rate without radical resection. The treatment effect of hepatic resection for T2 GBC is still controversial [14, 15]. Our study demonstrated that the survival rate in T2 GBC patients who underwent LN dissection with hepatectomy was not superior to that in patients who underwent LN dissection without hepatectomy, regardless of the tumor location. Park et al. [10] conducted a similar study and concluded that hepatic resection of T2b GBC did not affect long-term survival. In contrast, Lee et al. [9] reported that hepatic resection had a treatment effect on survival in T2b GBC, but not in T2a GBC. A multicenter study by Lee et al. [16] also showed that LN dissection without hepatectomy is a suitable treatment for T2a GBC, but not for T2b GBC.

The optimal extent of hepatic resection for T2 GBC has not been clearly determined. Although several clinical guidelines recommend segmentectomy IVb/V or non-anatomical liver resection with 2-cm margins around the GB bed [4–6], the German guidelines recommend non-anatomical liver resection with 3-cm margins around the GB bed [17]. The Korean guidelines recommend non-anatomical liver resection with a margin of 2–3 cm [4]. At our institute, we performed non-anatomical liver resections with 3-cm margins from the cystic plate.

Hepatic resection for GBC has the following three purposes: (1) to achieve a negative resection margin due to direct invasion of the liver from the GB bed, (2) to prevent recurrence near the GB bed due to micrometastasis of GBC near the GB bed, and (3) to prevent potential invasion of the hepatoduodenal ligament via en bloc resection of Glisson’s sheath of the right liver [15, 18, 19].

However, direct hepatic invasion of GBC was classified as pT3 GBC and extended surgical resection, including hepatectomy, is needed to achieve curative resection in these cases. T2b GBC is located on the hepatic side and can spread to the liver without penetrating the serosa; hepatectomy is recommended to achieve a tumor-free margin in such cases. In contrast, T2a GBC is located in the peritoneal side and is separated from the liver; hepatectomy is not needed to achieve a negative resection margin in such cases [9].

Another purpose of hepatic resection is to prevent recurrence through micrometastasis of GB cancer; this is based on several studies that frequently detected microscopic liver metastasis to the GB bed [8, 20, 21]. Some authors have reported that intrahepatic metastases are more frequent in T2b GBC patients, and direct drainage of the intrahepatic LNs is associated with T2b GBC [8, 22]. Moreover, the recurrence pattern of T2b GBC was more intrahepatic than that of T2a GBC in the current study. However, these recurrences of T2b GBC are systemic, and there was no evidence that partial hepatectomy prevents postoperative liver metastasis [10, 14, 15]. Partial hepatectomy, including wedge resection of the GB bed and segmentectomy, is not appropriate for en bloc resection of Glisson’s sheath of the right liver. To achieve en bloc resection of Glisson’s sheath of the right liver, right hepatectomy is necessary [19]. Jarnagin et al. [23] reported that GBC has a high incidence of distant metastases as a recurrence site. We also observed that T2b GBC has high incidence rates of distant metastasis and intrahepatic metastasis than T2a GBC (Fig. 1), suggesting that systemic treatments, such as adjuvant chemoradiotherapy, are needed to prevent systemic recurrence, particularly in patients with T2b GBC [8]. Wang et al. showed a survival benefit in higher stage (T2 or greater) or node-positive GBC patients who received adjuvant therapy [24]. Using the National Cancer Data Base, Kasumova et al. [25] recently found that adjuvant chemotherapy provides a survival benefit in T2 and T3 GBC patients. However, a meta-analysis by Ma et al. [26] revealed that patients with T3 or more advanced stage disease, node positivity, and margin positivity can benefit more from adjuvant therapy than patients with T2 GBC.

Recently, several studies have reported that tumor location is an important prognostic factor [8, 9]. Shindoh et al. [8] showed that T2a GBC was associated with a good prognosis compared with T2b GBC (5-year survival rate, 64.7% vs. 42.6%, *p* = 0.006). In the present study, T2a GBC patients showed better survival than T2b GBC patients (96.6% vs. 76%, *p* = 0.041). T2b GBCs drain directly into an intrahepatic venous or lymphatic route, while T2a GBCs usually drain into the pericholecystic route [27]. The anatomic differences in drainage routes between T2a and T2b GBC may explain the difference in survival [8].

LN metastasis is an independent significant prognostic factor for survival in patients with GBC [28–30]. LN metastasis occurred frequently in T2 GBC, with a high incidence of 62%; thus, LN dissection was essential for curative resection [29, 31–34]. Our study also showed that LN metastasis was the only independent prognostic factor for long-term survival in T2 GBC patients, while the extent of surgical resection did not impact survival.

In our study, we performed EHBD in 7 cases. Before 2000, in cases of tumors located near the infundibulum or cystic duct, we performed EHBD to achieve complete LN and radical resection. Patients with cystic duct or bile duct involvement were excluded from this study. However, after 2000, we changed the surgical treatment method and resected the extrahepatic bile duct only for GBC with extrahepatic bile duct involvement. This was because EHBD is not associated with any beneficial survival effects and is not recommended by the Korean guidelines [4].

We recommended adjuvant chemotherapy for patients with node-positive T2 GBC. Studies have reported that adjuvant chemotherapy is associated with improved survival in patients with LN metastasis [24, 35]. In the HR group, the number of patients with lymph node metastasis was significantly more than that in the Non-HR group. Hence, adjuvant chemotherapy was administered in the HR group with significantly more frequency than that in the Non-HR group.

This study has some limitations, including the small number of enrolled cases and retrospective study design; thus, prospective studies with a large number of patients are needed to verify our findings.

## Conclusion

Extended cholecystectomy without hepatic resection achieves favorable survival in both patients with T2a GBC and T2b GBC. Our findings show that lymphadenectomy alone without hepatic resection is a sufficient treatment for T2 GBC, regardless of tumor location. The need for hepatic resection for T2 GBC should be evaluated in further studies.

## Abbreviations

AJCC: American Joint Committee on Cancer
DFS: Disease-free survival
EHBD: Extrahepatic bile duct resection
GBC: Gallbladder cancer
LN: Lymph node
OS: Overall survival
